# Evaluation of a Quantitative Dual-Target EBV DNA Test on a Fully Automated Molecular Testing System

**DOI:** 10.1128/jcm.00518-23

**Published:** 2023-07-11

**Authors:** Marc Lütgehetmann, Eliseo Albert, Aaron Hamilton, Daniel Jarem, Susanne Pfefferle, Heinz Stucki, David Navarro

**Affiliations:** a University Medical Center Hamburg-Eppendorf (UKE), Hamburg, Germany; b Hospital Clínico Universitario, Fundación INCLIVA, School of Medicine, Valencia, Spain; c Roche Molecular Systems, Pleasanton, California, USA; d Roche Diagnostics International, Rotkreuz, Switzerland; Cepheid

**Keywords:** genotype, herpesvirus 4, humans, limit of detection, lymphoproliferative disorder, molecular diagnostic techniques, reference standards, viral load, cobas 6800, cobas 8800, cobas EBV test

## Abstract

The measurement of Epstein–Barr virus (EBV) deoxyribonucleic acid (DNA) is key to diagnosing and managing EBV-associated complications in transplant recipients. The performance of the new Conformité Européenne (CE) and Food and Drug Administration (FDA)-cleared quantitative Roche cobas EBV real-time PCR assay was determined by using EDTA-plasma dilution panels and clinical samples that were spiked with either the World Health Organization’s EBV international standard or high-titer EBV lambda stock. Correlation with the Abbott Realtime EBV assay was assessed in clinical specimens and conducted at two independent laboratories. An *in silico* analysis revealed that the dual-target test (*EBNA1* and *BMRF2*) was 100% inclusive for the known diversity of EBV. The overall limit of detection (LoD) was 16.6 IU/mL for genotype 1 (GT1). GT2 LoD was verified at 18.8 IU/mL. The linear ranges were from 1.40 × 10^1^ to 2.30 × 10^8^ IU/mL and from 2.97 × 10^1^ to 9.90 × 10^7^ IU/mL for GT1 and GT2, respectively. Accuracy was confirmed across the linear range (mean difference not exceeding ±0.18 log_10_). Precision was not influenced by the factors analyzed (standard deviation of 0.02 to 0.17 log_10_), including the presence of potentially interfering endogenous or exogenous substances. Plasma samples were stable under several conditions (variable time points, storage, and freeze/thaw cycles). In clinical EBV DNA-positive samples, correlation between the cobas EBV test and the comparator was high (*n* = 126 valid results; R^2^ = 0.96) with a 0.1 mean log_10_ titer difference. The cobas EBV test is an accurate, sensitive, specific, and reproducible assay for the detection of EBV DNAemia in plasma. In general, high levels of automation and calibration to the international standard will lead to improvements in the harmonization of quantitative EBV DNA test results across laboratories.

## INTRODUCTION

Epstein-Barr virus (EBV) is estimated to infect approximately 90% of adults worldwide and is the main cause of infectious mononucleosis (1). In recipients of solid organ transplants and hematopoietic cell transplants, viral infections are frequent causes of morbidity and mortality (2, 3), and infection with EBV is associated with the development of posttransplant lymphoproliferative disorder (PTLD) in such immunosuppressed patients (4). EBV DNAemia is routinely monitored in transplant recipients who are at high risk of PTLD as well as in those who are undergoing therapy for PTLD (5). Indeed, high levels of EBV deoxyribonucleic acid (DNA) in asymptomatic patients can serve as a marker for clinicians to initiate preemptive therapy and halt disease progression (5). In patients undergoing monitoring of EBV DNAemia, serial quantitative nucleic acid tests (NATs) can be used to indicate the need for potential treatment. As such, current guidelines from the European Society for Blood and Marrow Transplantation recommend the serial quantitative measurement of EBV DNAemia, post transplantation (6).

A wide range of commercial assays and laboratory-developed tests with which to assess EBV DNAemia are available, and results may be reported as copies/mL, copies/μg DNA, copies/number of cells, or IU/mL (7). The diversity in PCR methods and the lack of technical standardization are barriers to the optimal use of quantitative PCR assays, and they hinder comparisons of results between clinical laboratories, which is particularly important if patient testing is transferred to other laboratories. The World Health Organization’s (WHO) international standard for EBV (WHO EBV IS) (8) was introduced to address variation due to assay calibration and has been shown to offer some improvement in the variability of results (9), although a high level of variability has still been reported, dependent upon the testing protocol utilized (10).

This paper presents the results of technical performance verification studies for the new molecular, dual-target, Food and Drug Administration (FDA)-cleared, quantitative cobas EBV test, which runs on a fully automated high-throughput molecular testing system (cobas 6800/8800 System) (11), as well as the method correlation with an alternative Conformité Européenne (CE)-IVD-marked EBV test.

## MATERIALS AND METHODS

### *In silico* analysis of the cobas EBV assay to assess assay design inclusivity and exclusivity.

All available EBV sequences, including at least one of the two assay target genes, namely, *EBNA1* and *BMRF2*, were downloaded from the National Center for Biotechnology Information (NCBI). The analysis was performed in August of 2020 and was updated to May of 2021. Sequences were aligned to the assay oligonucleotides of each of the two gene targets to look for mismatches. Roche proprietary bioinformatic algorithms that were specific to the master mix were utilized to predict the potential effects of any detected mismatches (for more details, see [12]). We screened for the presence of mismatches that may impact oligonucleotide performance, where impact was defined as a cycle threshold increase that was greater than five cycles and/or a probe melting temperature of <65°C. The *in silico* exclusivity analysis utilized similar methods (accessed in September of 2020; for more details, see [12]) and screened for cross-reactivity with non-EBV species by using sequences found in the NCBI database that may have similar sequence homology, including related herpesviruses as well as other viral, bacterial, or human sequences.

### Quantitative EBV assays: cobas EBV test and Abbott RealTime EBV assay.

The cobas EBV test was performed on either a cobas 6800 System or a cobas 8800 System (13). As these systems are fully automated molecular analyzers, all steps, including primary sample handling, DNA extraction, and PCR amplification, were performed in the same system without further manual intervention. The comparator assay, namely, the CE-IVD-approved (but not FDA-cleared) Abbott RealTime EBV assay, was performed on an m2000 RealTime System (m2000sp instrument for DNA extraction and 2000rt for PCR amplification), according to the manufacturer’s instructions (14).

### Limit of detection (LoD).

The WHO EBV IS, NIBSC code 09/260, strain B95-8 (8) titer, which was confirmed in-house by using an Abbott RealTime EBV assay, was spiked into a single plasma pool that was composed of plasma from 27 anti-EBV IgG-negative and IgM-negative donors, and it was used to create a six-point dilution series for the determination of the LoD (95%) for EBV GT1. The dilution series ranged from 2.5 to 50 IU/mL (three levels below the expected LoD, one level at the expected LoD, and two levels above the expected LoD) and included a negative set. The series was assessed with three different cobas EBV kit lots, across 3 days, on three systems, generating 189 replicates per concentration level.

An EBV-positive cell culture supernatant of GT2, namely, strain Jiyoye (GT2-J) from the American Type Culture Collection, the titer of which was confirmed in-house by using the Abbott RealTime EBV assay, was spiked into a single plasma pool that was composed of plasma that was derived from anti-EBV IgG and IgM EBV-negative donors, and it was used to create a three-point dilution series for the determination of the LoD (95%) for EBV GT2. Each series consisted of three concentration levels, namely, 9.4 IU/mL, 18.8 IU/mL, and 28.2 IU/mL, representing 0.5×, 1.0×, and 1.5× the LoD for GT1 (derived from the previous experiment, value taken from the least sensitive kit). Testing used three different cobas EBV kit lots and was conducted across 3 days, generating 63 replicates per concentration level.

### Linearity.

For EBV GT1, a commercial GT1-positive clinical sample (estimated titer 1.42 × 10^5^ IU/mL; Aalto Bio Reagents) was used to prepare six panel members, covering the lower and intermediate levels of the linear range (the sample was not sufficiently concentrated for the upper levels of the linear range). A high-titer EBV lambda DNA stock sample (phagemid; Roche Molecular Systems, Branchburg, USA) was used to prepare 11 panel members that spanned the entire linear range. The use of these two materials allowed for the creation of a 17-point dilution series that spanned the expected linear range from 1.5 × 10^1^ to 2.0 × 10^8^ IU/mL. The study was conducted using three kit lots, and the results presented are for both sample types (phagemid and clinical) and kit lots combined. A total of 36 replicates were analyzed per concentration level.

For EBV GT2, linearity was assessed by using an eight-point dilution series that spanned the expected linear range from 3.00 × 10^1^ to 1.00 × 10^8^ IU/mL. The panel was prepared via the serial dilution of the EBV cell culture supernatant of EBV GT2, strain Jiyoye, in pooled EDTA-plasma. The viral load was determined by using an Abbott RealTime EBV assay. A total of 12 replicates were analyzed per concentration level.

The linear range was defined as the concentration range for which the deviation of the predicted log_10_ titer of the best fitting higher model and the predicted log_10_ titer of the linear regression were within ±0.3 log_10_.

### Lower limit of quantitation (LLoQ).

The LLoQ provides the lowest concentration level within the observed linear range that is not lower than the determined LoD (95% hit rate) and still meets quantitative parameters for accuracy and precision. The acceptance criterion for this study was that both the total analytical error (TAE) and the “difference between measurements in standard deviation (SD)” values had to meet the acceptance criterion of ≤1 log_10_, where TAE = bias + 2SD, as per the Clinical and Laboratory Standards Institute (CLSI) EP-17A2 guideline (15), and the difference between measurements = 2 × √2 × SD.

### Accuracy and precision.

The accuracy of the cobas EBV test was determined by using a dilution of the WHO EBV IS spiked in EDTA-plasma. Each concentration level (200 IU/mL, 2,000 IU/mL, and 20,000 IU/mL) was measured with 62 replicates and tested using three kit lots. Accuracy was calculated by subtracting the log_10_ nominal titer from the mean log_10_ observed titer. Results are presented for the kit lots combined.

For precision, a seven-member dilution series spanning the linear range 6 × 10^1^ to 5 × 10^7^ IU/mL was prepared by using the aforementioned EBV GT1-positive material (phagemid) spiked in EDTA-plasma. Precision was calculated based on results generated over 12 days, three kit lots, and three cobas 6800 Systems to produce 72 replicates per panel member.

### Exogenous and endogenous interference, cross-reactivity, and stability.

For details, please see the Supplemental Material, including Table S1 (list of organisms used for the cross-reactivity analysis) and Fig. S1 (storage conditions for the stability assessment).

### Clinical samples method correlation.

For the method correlation, approximately 250 remnant anonymized EDTA-plasma samples from EBV-positive patients were available after standard-of-care molecular EBV DNA testing (Valencia or Hamburg). The samples were stored at −20°C for up to 6 months. The use of remnant anonymized samples for the method correlation study was waived by the ethics committees of the Hospital Clínico Universitario, Fundación INCLIVA, and the ethics committees of the Hansestadt Hamburg (Germany) prior to the initiation of the study. The study was conducted in compliance with the International Conference on Harmonization Good Clinical Practice Guidelines and local regulations.

Bland-Altman and Deming linear regression were employed to determine quantitative agreement between the assays. Each sample was measured once per method and was included in the analysis only if it met the protocol inclusion criteria, meaning that it had a valid quantitative result from both the cobas EBV test and the RealTime EBV assay as well as a value above the LLoQ for both assays. Protocol deviations (i.e., inadvertent replicates or the wrong diluent being used) were excluded from the analysis.

### Statistical analysis.

The statistical analysis included the calculation of the LoD by using a probit analysis (including 95% confidence intervals [CI]) and a hit rate analysis, the SD of the log_10_ titers, and a Bland-Altman and Deming or polynomial regression analysis, including Grubbs’ outlier test.

Testing was conducted using a cobas 6800/8800 System configuration version CI-Serie 1.13, including software version 01.03.08 and EBV configuration baseline 2.1. Subsequent calculations were done using Mastersession EBV V0010 (EBV configuration baseline 3.0). Additional data handling was done using the Growth Curve Analysis Tool version 10.7.2.0 as well as SAS JMP (version 12.1.0; JMP Statistical Discovery, Cary, NC, USA).

### Study sites.

The *in silico* evaluation of assay design inclusivity was performed at Roche Molecular Systems, Inc., Pleasanton, CA, USA. Technical performance testing was carried out at Roche Diagnostics International, Rotkreuz, Switzerland. Method correlation was conducted with clinical samples at two independent laboratories: University Medical Center Hamburg-Eppendorf (UKE) in Germany and Hospital Clínico Universitario, Fundación INCLIVA, School of Medicine in Valencia, Spain.

### Data availability.

The data reported herein were used for obtaining FDA and CE clearance and are partly shown in the instructions for use (13). Data are available upon reasonable request.

## RESULTS

The number of samples utilized per analysis is provided in Fig. S2.

### *In silico* analysis of design inclusivity and exclusivity.

The cobas EBV test targets the *EBNA-1* (Epstein–Barr nuclear antigen 1) and *BMRF2* genes. Of the 1,162 sequences that were downloaded from NCBI, 1,158 included assay target 1, whereas 1,151 sequences included assay target 2. 1,147 sequences contained both targets. 100% of the sequences containing both targets were free of mismatches in one or both target oligonucleotide set binding positions. The two target genes were free of mismatches in 96% (target 1) and 98.3% (target 2) of the downloaded sequences. All sequences with a mismatch in one target were a perfect match in the second oligonucleotide set. A subset of observed mismatches could have an effect on oligonucleotide performance for one target only. No sequence was predicted to impact the dual-target assay as a whole, providing 100% predicted inclusivity for the EBV GT1 and EBV GT2 isolates. The assay design was also shown to be exclusive, with no non-EBV sequences predicted to be detected by the assay.

### LoD, LLoQ, and linearity.

The probit analysis revealed that the LoD for GT1 was 16.6 IU/mL (95% CI, 14.3 to 20.0) and a ≥95% hit rate was observed at 20.0 IU/mL (Table S2). The LoD for the least sensitive kit lot (kit lot 1) was 18.8 IU/mL (95% CI, 14.5 to 27.5). The LoD for GT2 was verified at 18.8 IU/mL with a hit rate analysis (Table S3).

**FIG 1:**
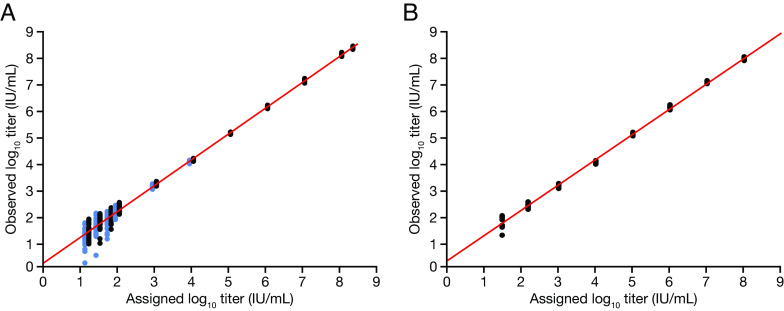
Linear assessment for EBV GT1 and GT2. The regression line is shown in red. 36 replicates were tested per concentration for GT1, and 12 replicates were tested per concentration for GT2. (A) One clinical specimen was used to prepare six panel members, covering the intermediate and lower levels of the linear range (data points indicated in blue), whereas other panel members were prepared by using a EBV lambda phagemid (data points indicated in black). (B) Panel members prepared by using an EBV cell culture supernatant of EBT GT2 (strain Jiyoye). EBV, Epstein–Barr virus; GT, genotype.

The linear range of the cobas EBV test was determined to be 1.40 × 10^1^ to 2.30 × 10^8^ IU/mL and 2.97 × 10^1^ to 9.90 × 10^7^ IU/mL for GT1 and GT2, respectively (Fig. 1 A and B). The LLoQ was calculated to be 35 IU/mL. Based on the LLoQ and the determined linear range, the linear measurement range of the test was set to 35.0 to 1 × 10^8^ IU/mL.

### Accuracy and precision.

The mean difference between the observed and nominal titer did not exceed ±0.18 log_10_ IU/mL for any of the tested concentrations (Table S4). Precision was comparable across all kit lots and concentration levels, with a SD between 0.02 and 0.17 log_10_ (Table S5).

### Exogenous/endogenous interference and cross-reactivity.

The mean log_10_ titer of each of the positive EBV samples containing an exogenous/endogenous substance was within ±0.5 log_10_, compared to the control (Table S6 and S7). With respect to sensitivity and specificity, none of the tested substances interfered with the cobas EBV test. All of the EBV-positive specimens, whether spiked with a potential cross-reactant pool or a single interferent, remained positive (Table S8). The mean Δlog_10_ titers were within ±0.05 log_10_ of that of the EBV positive control. Similarly, all EBV-negative specimens, whether spiked with or without each cross-reactant pool or single interferent, remained negative for EBV.

### Stability of EDTA-plasma samples for the analysis of EBV.

The mean EBV log_10_ titer differences across the various conditions (collection tube, storage time, and temperature) ranged between −0.02 and −0.29 log_10_ (Table S9).

### Clinical samples method correlation.

**FIG 2:**
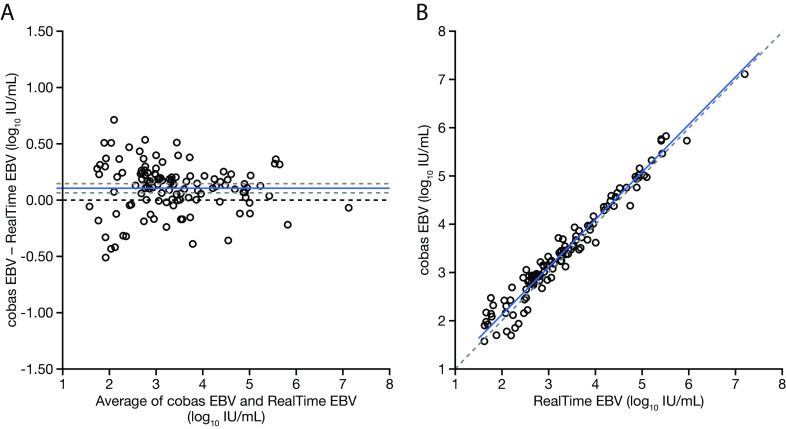
Quantitative agreement of the Roche cobas EBV test and the Abbott RealTime EBV assay. (A) A Bland-Altman plot demonstrated a mean viral load difference of +0.1 log_10_ IU/mL. The bias (blue solid line), 95% confidence intervals (gray dashed lines), and zero line (black dashed line) are shown. (B) Deming regression resulted in a regression line of *y* = 0.99*x* + 0.14, with 95% confidence intervals of the slope (0.95 to 1.03) and intercept (−0.001 to 0.3). The regression line (blue solid line) and zero line (gray dashed line) are shown. No outliers were detected. EBV, Epstein–Barr virus; IU, international units.

The concentration of the EBV DNA that was detected ranged from 36 to 1.27 × 10^7^ IU/mL, as measured by a cobas EBV test, and the median EBV DNA level was 1.35 × 10^3^ IU/mL. With all of the clinical specimens having been sourced from EBV-positive patients, the positive percent agreement between the cobas EBV test and the RealTime EBV assay was 97.4%. The overall percent agreement between the two tests was 94.4% (152/161 samples were qualitatively concordant). Nine samples were discordant negative, and all of these had low viral loads, with eight of them being unquantifiable (<35 IU/mL cobas EBV test; <40 IU/mL RealTime EBV assay) and one of them having a reported viral load of 57.6 IU/mL, as measured by a cobas EBV test. The mean log_10_ titer difference between the viral load results from the cobas EBV test and the RealTime EBV assay across the entire linear range was found to be 0.1 log_10_ IU/mL with a two-sided 95% CI of 0.06 to 0.14 log_10_ IU/mL (Fig. 2A). A strong correlation was observed (R^2^ = 0.96) and the slope of the regression line was 0.99 (95% CI, 0.95 to 1.03) (Fig. 2B). No outlier samples were identified by Grubbs’ outlier test.

## DISCUSSION

Sensitive and accurate quantitative EBV nucleic acid amplification tests play a critical role in the management of patients who are at high risk of severe EBV infection or reactivation and who may benefit from the early initiation of treatment (5). However, the heterogeneity of the methodologies in use for determining EBV DNAemia, as well as the lack of technical standardizations between labs, hinders the comparability of quantitative results. Consequently, current guidelines and clinical practice require the monitoring of EBV DNAemia trends, rather than having clear decision points (16–18).

Here, we present the results of the technical validation of the new cobas EBV test for use on automated cobas 6800/8800 Systems by using EDTA-plasma specimens. The commercially available assay has a dual-target design that detects the EBV single copy genes *EBNA1* and *BMRF2*. A bioinformatic evaluation of the full and partial EBV genome sequences that were available from the NCBI database (as of August of 2020) indicated that the assay is 100% inclusive, thereby allowing for accurate detection and quantitation within the known genetic diversity of EBV. The importance of the dual-target approach to safeguard against sporadic circulating mutations in the primer and probe regions, as well as mutations that may arise in the future, is one of the lessons learned from HIV (19) and SARS-CoV-2 qPCR design (20).

Analytically, the assay demonstrated an LoD of 18.8 IU/mL for both EBV GT1 and GT2. Some commercial EBV tests report their LoD in copies per mL (21, 22), which precludes the evaluation of the cobas EBV test in comparison with these molecular tests; however, the cobas EBV test has a lower LOD than other EBV plasma-based molecular EBV DNA assays that report in IU/mL, such as the comparator used in this study (RealTime EBV assay, 48.9 IU/mL) (23) and the EBV assay by Altona Diagnostics (195 IU/mL) (24), although the clinical relevance of this difference at such low concentrations is unclear.

The cobas EBV test was linear across a wide range of dilutions and showed strong concordance with the WHO EBV IS (maximal divergence of 0.12 log_10_ IU/mL). The intrarun precision was high (low variability) across the linear range (pooled SD from 0.04 at 5.40 × 10^7^ IU/mL to 0.16 at 6.48 × 10^1^ IU/mL), demonstrating excellent reproducibility. This can be attributed mainly to the fully automated nature of the sample preparation, purification, and amplification on the cobas platform (11). Furthermore, high precision and consistency were observed across different operators and time points (i.e., different runs on different days), indicating that results are not substantially impacted by varying conditions in diagnostic practice.

Pre-analytically, the evaluation of the sample stability showed that whole blood collected in EDTA-plasma tubes may be stored or transported for up to 24 h at 2 to 30°C prior to plasma preparation without significant impact on EBV titers. For storage times longer than 24 h, the samples should be separated after 24 h in EDTA-plasma, after which they can be stored for an additional 6 days at 2 to 30°C, followed by another 6 days at 2 to 8°C or 6 months at −15 to −80°C without an effect on the measurement of EBV DNA.

The correlation of the cobas EBV test with the comparator assay, reported in international units, was performed by using EBV DNA-positive clinical specimens. The mean log_10_ titer difference between the viral load results from the cobas EBV test and those from the RealTime EBV assay across the entire linear range was found to be 0.1 log_10_ IU/mL with a strong correlation (R^2^ = 0.96), highlighting the comparable performance of the two automated quantitative EBV DNA qPCR tests in clinical samples. In a real-world setting, it is not uncommon for patients to travel long distances to receive transplants (25, 26), and, upon returning home, frequent testing can become burdensome, often requiring samples to be sent back to the original laboratories or re-baselining at a new location. In this context, the use of such assays can remove some of these hurdles and provide a positive impact on patients and patient management.

## CONCLUSIONS

The data presented in this study demonstrate the high sensitivity, accuracy, and consistency of the cobas EBV test for the detection of EBV DNA in plasma, featuring broad coverage and inclusivity due to the dual-target design and comparable clinical performance with other available qPCR solutions. In general, the data presented here support that any assay with high levels of automation and calibration to the international standard will lead to improvements in the harmonization of quantitative EBV DNA test results across laboratories. This can positively impact patient care and might be a basis for universal therapeutic viral load thresholds in the future.
