# Supplementary material for: Evaluation of a Quantitative Dual-Target EBV DNA Test on a Fully Automated Molecular Testing System
Source: J Clin Microbiol. 2023 Jul 11;61(8):e00518-23. doi: 10.1128/jcm.00518-23 (PMC10446858; doi:10.1128/jcm.00518-23)
Supplement: Supplemental file 1 — Supplemental material. Download jcm.00518-23-s0001.pdf, PDF file, 0.3 MB [file jcm.00518-23-s0001.pdf]

Technical performance evaluation of an EBV DNA quantitative test in plasma samples on the cobas 6800/8800 systems

Marc Lütgehetmann[a]#;

Eliseo Albert[b];

Aaron Hamilton[c];

Daniel Jarem[c];

Susanne Pfefferle[a];

Heinz Stucki[d];

David Navarro[b]

[a]University Medical Center Hamburg-Eppendorf (UKE), Hamburg, Germany

[b]Hospital Clínico Universitario, Fundación INCLIVA, School of Medicine, Valencia, Spain

[c]Roche Molecular Systems, Pleasanton, CA, USA

[d]Roche Diagnostics International, Rotkreuz, Switzerland

Performance evaluation of the cobas EBV test (max 54 characters with spaces; 44/54)

#Address correspondence to Marc Lütgehetmann, mluetgeh@uke.de

SUPPLEMENTAL MATERIALS

Supplemental methods

Exogenous and endogenous interference

The influence of exogenous substances (24 different commercially available drugs commonly administered to virus-infected and transplant patients) and endogenous interferents (albumin, conjugated bilirubin, unconjugated bilirubin, hemoglobin, human DNA, or triglycerides), as well as solvent controls, on the performance of the cobas EBV test was evaluated at 5× LLoQ.

An EBV-positive sample was spiked into individual EBV-negative EDTA-plasma samples at a concentration of 150 IU/mL EBV DNA (assigned using the comparator test). The testing concentration of each active component (exogenous) was at 3× plasma peak level (3× C_{max}). Levels of albumin, bilirubin (conjugated and unconjugated), hemoglobin, and triglycerides for the spiked samples tested were those recommended by the CLSI (Clinical and Laboratory Standards Institute) guideline EP07-A2 (1). Since human DNA levels are not listed in the CLSI guidelines, the average of published maximum plasma DNA levels (2 mg/L) was used. As a positive spike control, each donor specimen was tested without interferent. As a negative spike control, each donor specimen was tested without the interferent and without EBV. Solvent controls were tested for each solvent used (dimethylsulfoxide, ethanol, and phosphate-buffered saline).

Cross-reactivity

Cross-reactivity of the cobas EBV test was evaluated by testing with 35 different microorganisms, including 17 viruses, 15 bacterial strains, and three fungal isolates, grouped into seven cross-reactant pools and as a single interferent (hepatitis C virus) (**Supplemental Table 1**). All cross-reactant pools and the single interferent were spiked with either EBV-negative or EBV-positive pooled EDTA-plasma with a final concentration of 150 IU/ml. The study was conducted over 2 days, testing six replicates per pool/single interferent for EBV-positive and EBV-negative pools, using two cobas 6800/8800 systems and three kit lots. Results are presented for all kit lots combined.

Stability of EDTA-plasma samples for analysis of EBV

The effect of storage medium/duration/temperature, and the influence of freeze/thaw cycles, on the cobas EBV test was determined. Five EBV-positive clinical specimens were used to spike 10 unique individual EBV-negative donors (final EBV concentration of 150 IU/mL). Five of these 10 specimens were collected in BD Vacutainer® PPT™ (Plasma Preparation Tube [PPT]) whilst the other five specimens were collected in BD lavender top tubes (lavender top). The storage conditions assessed are presented in **Supplemental Figure 1**. In summary, whole blood was collected in PPT or lavender top tubes (sterile tubes using EDTA as the anticoagulant) and stored for 24 hours at 2–25°C prior to plasma preparation. Upon separation, plasma samples were stored for 24 hours at 2–30°C in primary or secondary tubes, followed by storage in primary or

secondary tubes for up to 6 days at 2–8°C. The effect of long-term storage on plasma samples in secondary tubes was assessed at 6 months at -15– -80°C.

For determination of stability over freeze/thaw cycles, at timepoint T0, the separated plasma was transferred into secondary tubes and underwent four freeze/thaw cycles at either -15°C or -80°C.

Results are presented as the mean log_{10} titer difference (in comparison to the T0 reference titer in either lavender top tube or PPT as relevant).

Supplemental results

Supplemental Figure 1: Storage conditions to evaluate whole blood and plasma stability

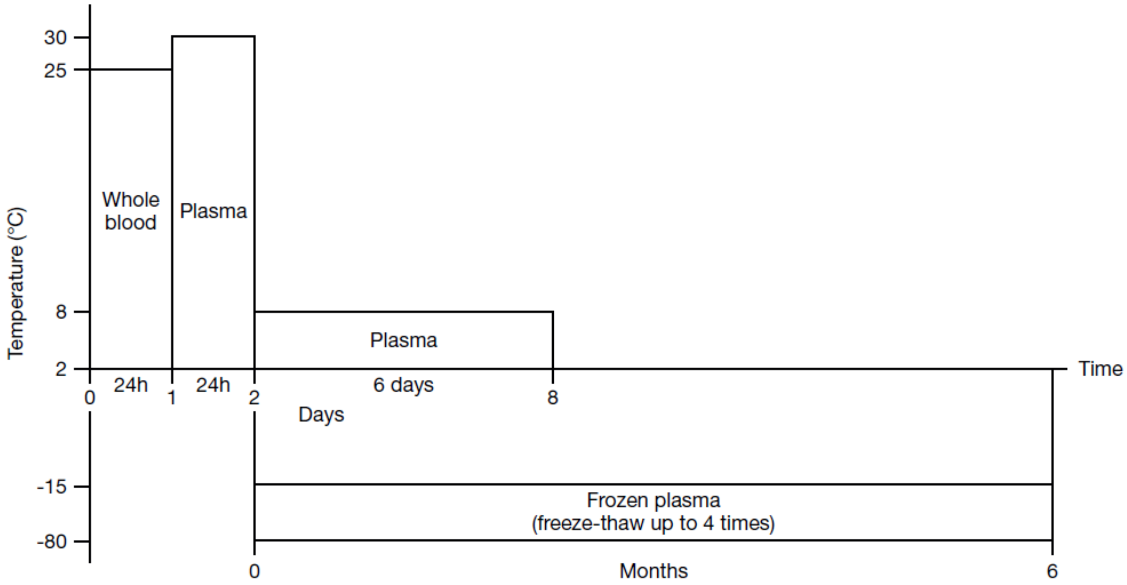

h, hours

Supplemental Figure 2: Flowchart summarizing samples utilized per analysis with details of any exclusions

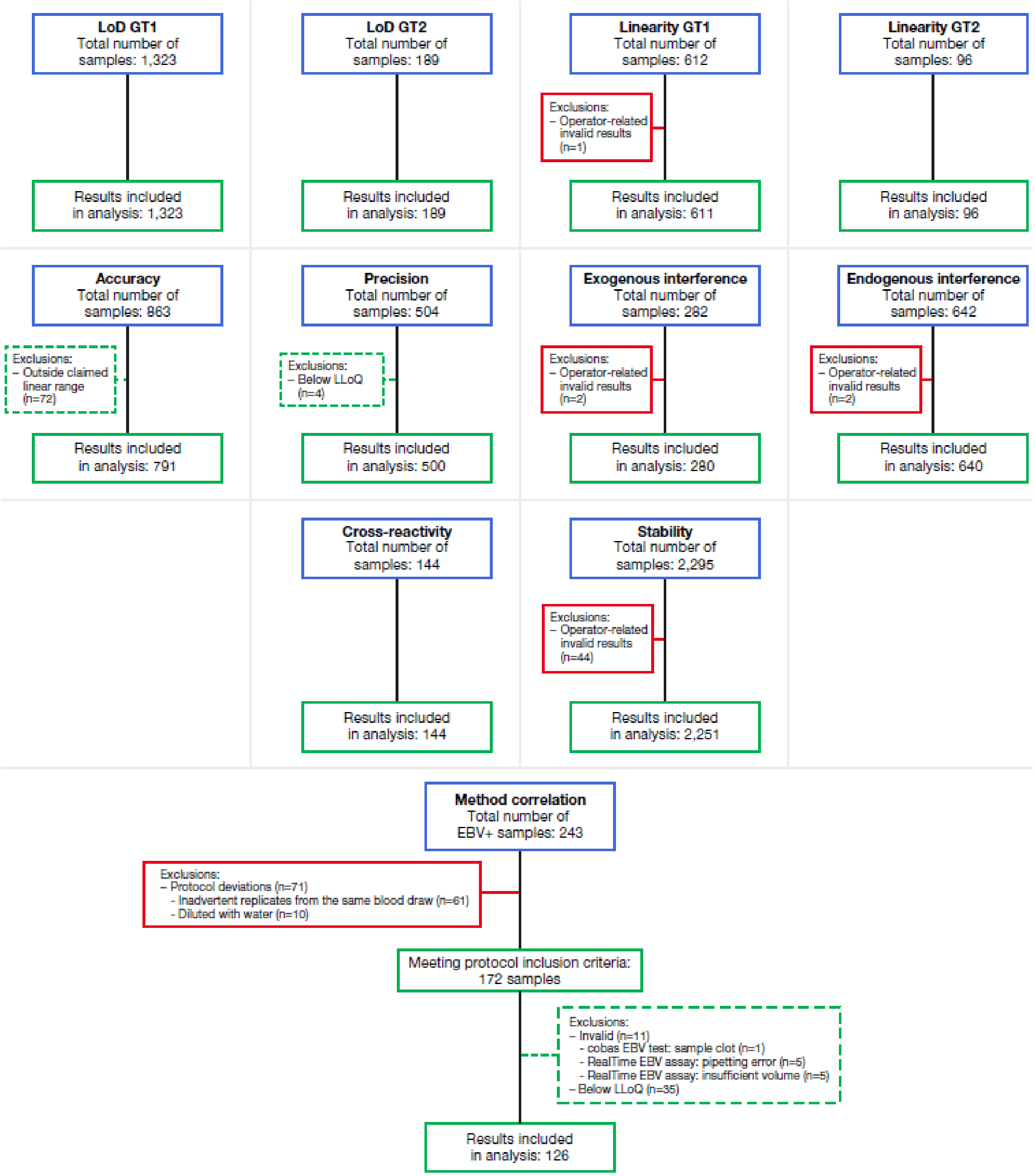

EBV, Epstein–Barr virus; GT, genotype; LLoQ, lower limit of quantitation; LoD, limit of detection.

Supplemental Table 1: List of microorganisms, grouping in cross-reactant pools and concentration

Pool	Microorganisms	Concentration tested	Vendor
**1**	Herpes simplex virus type I	1.00×10^{6} copies/mL	ZeptoMetrix
	Herpes simplex virus type II	1.00×10^{6} copies/mL	ZeptoMetrix
	Human herpesvirus type 6	1.00×10^{6} copies/mL	ZeptoMetrix
	Human herpesvirus type 7	1.00×10^{6} copies/mL	ZeptoMetrix
	Human herpesvirus type 8	1.00×10^{6} copies/mL	ZeptoMetrix
**2**	Adenovirus type 5	1.00×10^{6} $TCID_{50}$/mL	American Type Culture Collection
	Candida albicans	1.00×10^{6} CFU/mL	ZeptoMetrix
	Chlamydia trachomatis	1.00×10^{6} IFU/mL	American Type Culture Collection
	Clostridium perfringens	1.00×10^{6} cells/mL	Deutsche Sammlung von Mikroorganismen und Zellkulturen
	Cytomegalovirus	1.00×10^{6} IU/mL	Biomex
**3**	*Enterococcus faecalis*	1.00×10^{6} CFU/mL	ZeptoMetrix
	Escherichia coli	1.00×10^{6} cells/mL	Deutsche Sammlung von Mikroorganismen und Zellkulturen
	Hepatitis B virus	1.00×10^{6} IU/mL	ZeptoMetrix
	Human immunodeficiency virus type 1	1.00×10^{6} IU/mL	ZeptoMetrix
	Human immunodeficiency virus type 2	$1.00x10^{6}$ IU/mL	AccuType™
**4**	*Klebsiella pneumoniae*	1.00×10^{6} CFU/mL	ZeptoMetrix
	Listeria monocytogenes	1.00×10^{6} CFU/mL	ZeptoMetrix

	Mycobacterium avium	1.00×10^6 CFU/mL	Roche Diagnostics, Penzberg
	Mycoplasma pneumoniae	1.00×10^6 CFU/mL	ZeptoMetrix
	Neisseria gonorrhoeae	1.00×10^6 CFU/mL	ZeptoMetrix
**5**	Parvovirus B19	1.00×10^6 IU/mL	ZeptoMetrix
	Propionibacterium acnes	1.00×10^6 CFU/mL	ZeptoMetrix
	Salmonella enterica	1.00×10^6 CFU/mL	ZeptoMetrix
	Simian virus 40	1.00×10^6 $TCID_{50}$/mL	American Type Culture Collection
	Staphylococcus aureus	1.00×10^6 CFU/mL	ZeptoMetrix
**6**	*Staphylococcus epidermis*	1.00×10^6 CFU/mL	ZeptoMetrix
	Streptococcus pyogenes	1.00×10^6 cells/mL	Deutsche Sammlung von Mikroorganismen und Zellkulturen
	Streptococcus pneumoniae	1.00×10^6 CFU/mL	ZeptoMetrix
	Varicella zoster virus	1.00×10^6 copies/mL	ZeptoMetrix
	Aspergillus niger	1.00×10^6 CFU/mL	Roche Diagnostics, Penzberg
**7**	*Cryptococcus neoformans*	1.00×10^6 cells/mL	Roche Diagnostics, Penzberg
	Human papilloma virus	1.00×10^6 cells/mL	BioCollections Worldwide
	Human polyomavirus 2 (JV virus)	1.00×10^6 copies/mL	American Type Culture Collection
	Human polyomavirus 1 (BK virus)	1.00×10^6 IU/mL	Exact Diagnostics
**HCV**	Hepatitis C virus	3.00×10^5 IU/mL	Boca Bioloistics

CFU, colony forming unit; HCV, hepatitis C virus; IFU, infectious units; IU, international units; $TCID_{50}$, median tissue culture infectious dose.

Supplemental Table 2: Summary limit of detection for EBV GT1 (three kit lots combined) in EDTA-plasma

EBV DNA concentration (IU/mL)	Number of positives	Number of valid	% positives
50.0	189	189	100.0
35.0	188	189	99.5
20	186	189	98.4
10	159	189	84.1
5	110	189	58.2
2.5	72	189	38.1
0	0	189	0.0
**LoD by probit analysis (95% hit rate)**	**16.6 IU/mL 95% confidence range: 14.3–20.0 IU/mL**		
LoD by hit rate	20.0 IU/mL		

EBV, Epstein–Barr virus; EDTA, ethylenediaminetetraacetic acid; GT, genotype; IU, international units; LoD, limit of detection.

Supplemental Table 3: Summary of hit rates for EBV GT2 (strain Jiyoye) in EDTA-plasma

Conc. level	Conc. IU/mL*	Positive results	Total valid results	Hit rate	Upper one-sided 95% CI	Lower two-sided 95% CI	Upper two-sided 95% CI
1.5× LoD	28.2	63	63	100%	100	94.3	100
1.0× LoD	18.8	62	63	98.4%	99.9	91.5	100
0.5× LoD	9.4	58	63	92.1%	96.8	82.4	97.4

*The 1× LoD concentration was based on ≥95% probit concentration level of the least sensitive kit lot from the LoD study of GT1

CI, confidence interval; conc, concentration; EBV, Epstein–Barr virus; EDTA, ethylenediaminetetraacetic acid; GT, genotype; IU, international units; LoD, limit of detection.

Supplemental Table 4: Summary of accuracy results at the LLoQ, with WHO standard dilutions, and across the linear range

Nominal/assigned log_{10} titer (IU/mL)	Mean observed log_{10} titer (IU/mL)	Δ mean observed to nominal/assigned log_{10} titer	Lower 95% CI	Upper 95% CI	Test case
1.54	1.60	0.05	-0.01	0.12	Accuracy at LLoQ*
2.30	2.42	0.12	0.09	0.15	Accuracy of WHO EBV IS**
3.30	3.36	0.05	0.04	0.07	
4.30	4.34	0.04	0.03	0.05	
1.54	1.72	0.18	0.09	0.27	Accuracy across the linear range***
1.73	1.79	0.05	-0.03	0.14	
1.84	1.96	0.12	0.06	0.19	
1.96	2.09	0.14	0.09	0.19	
2.06	2.22	0.16	0.12	0.20	
2.96	3.04	0.09	0.07	0.11	
3.06	3.15	0.09	0.07	0.11	
3.96	4.00	0.05	0.04	0.06	
4.06	4.10	0.04	0.03	0.05	
5.06	5.12	0.06	0.05	0.07	
6.06	6.15	0.08	0.07	0.10	
7.06	7.14	0.07	0.06	0.09	

Note: Absolute values were taken for calculation and rounded to 2 decimal places

*Data from the LoD study

**Data from the Accuracy study

***Data from the Linearity study (dilution series of EBV GT 1 lambda DNA [phagemid] and clinical sample); 539 samples in total

CI, confidence interval; IU, international units; LLoQ, lower limit of quantitation; WHO EBV IS, World Health Organization Epstein–Barr virus international standard; Δ, difference.

Supplemental Table 5: Summary of precision as SD of log_{10} titer results from EDTA-plasma

Panel member	Assigned concentration (IU/mL)	Precision as SD (log_{10})			Pooled SD (log_{10})
		Kit lot 1	Kit lot 2	Kit lot 3	
PM01	5.40×10^{7}	0.03	0.04	0.04	0.04
PM02	1.08×10^{6}	0.02	0.03	0.02	0.02
PM03	1.08×10^{5}	0.02	0.02	0.03	0.02
PM04	1.08×10^{4}	0.04	0.02	0.03	0.03
PM05	1.08×10^{3}	0.05	0.05	0.05	0.05
PM06	1.08×10^{2}	0.17	0.18	0.15	0.17
PM07	6.48×10^{1}	0.17	0.17	0.13	0.16

EDTA, ethylenediaminetetraacetic acid; PM, panel member; IU, international units; SD, standard deviation.

Supplemental Table 6: Performance of the cobas EBV test in the presence of potential exogenous interferents

		Samples with interferent and spiked with EBV			Samples with interferent and without EBV	
**Exogenous substances**	**Testing concentration (3× C_{max})**	**Positive valid replicates/ valid replicates**	**Sensitivity %**	**Mean log_{10} titer difference<br><br>EBV(+) interferent - EBV(+) control (SD)**	**Negative valid replicates/valid replicates**	**Specificity %**
**Pool 1 (solvent: PBS)**						
Azathioprine	3 µg/mL	30/30	100	-0.18 (0.20)	10/10	100
Sulfamethoxazole	204 µg/mL					
Trimethoprim (TPM)*	6 µg/mL					
Cefotetan	711 µg/mL					

		Samples with interferent and spiked with EBV			Samples with interferent and without EBV	
**Exogenous substances**	**Testing concentration (3× C_{max})**	**Positive valid replicates/ valid replicates**	**Sensitivity %**	**Mean log_{10} titer difference<br><br>EBV(+) interferent - EBV(+) control (SD)**	**Negative valid replicates/valid replicates**	**Specificity %**
Cidofovir	60 µg/mL					
**Pool 2 (solvent: PBS)**						
Foscarnet sodium	1869 µM	30/30	100	-0.21 (0.19)	10/10	100
Piperacillin	894 µg/mL					
Tazobactam sodium*	102 µg/mL					
Prednisone	36 µg/mL					
Vancomycin	189 µg/mL					
**Pool 3 (solvent: DMSO)**						

		Samples with interferent and spiked with EBV			Samples with interferent and without EBV	
**Exogenous substances**	**Testing concentration (3× C_{max})**	**Positive valid replicates/ valid replicates**	**Sensitivity %**	**Mean log_{10} titer difference<br><br>EBV(+) interferent - EBV(+) control (SD)**	**Negative valid replicates/valid replicates**	**Specificity %**
Cyclosporine	5.4 µg/mL	30/30	100	-0.17 (0.20)	10/10	100
Everolimus	12 µg/mL					
Fluconazole	20.1 µg/mL					
Ganciclovir	27 µg/mL					
**Pool 4 (solvent: DMSO)**						
Mycophenolate mofetil	75 µg/mL	30/30	100	-0.17 (0.19)	10/10	100
Mycophenolic acid	111 µg/mL					

		Samples with interferent and spiked with EBV			Samples with interferent and without EBV	
**Exogenous substances**	**Testing concentration ($3\times C_{max}$)**	**Positive valid replicates/ valid replicates**	**Sensitivity %**	**Mean log_{10} titer difference<br><br>EBV(+) interferent - EBV(+) control (SD)**	**Negative valid replicates/valid replicates**	**Specificity %**
Valganciclovir HCI	16.8 µg/mL					
**Pool 5 (solvent: ethanol)**						
Sirolimus	0.045 µg/mL	30/30	100	-0.16 (0.20)	10/10	100
Tacrolimus	0.21 µg/mL					
**Pool 6 (solvent: PBS)**						
Letermovir	39 µg/mL	30/30	100	-0.20 (0.19)	10/10	100
Micafungin	49.2 µg/mL					
Acyclovir	16.8 µg/mL					

		Samples with interferent and spiked with EBV			Samples with interferent and without EBV	
**Exogenous substances**	**Testing concentration (3× C_{max})**	**Positive valid replicates/ valid replicates**	**Sensitivity %**	**Mean log_{10} titer difference<br><br>EBV(+) interferent - EBV(+) control (SD)**	**Negative valid replicates/valid replicates**	**Specificity %**
Clavulanate potassium	3 μg/mL					
**Pool 7 (solvent: PBS)**						
Ticarcillin disodium	972 μg/mL	30/30	100	-0.19 (0.20)	10/10	100

*Tested at >3× C_{max}

DMSO, dimethylsulfoxide; EBV, Epstein–Barr virus; PBS, phosphate-buffered saline; SD, standard deviation.

Supplemental Table 7: Performance of the cobas EBV test in the presence of potential endogenous interferents

		Samples with interferent and with EBV Target (~5× LLoQ, 150 IU/mL)			Samples with interferent and without EBV target	
**Endogenous substances**	**Test concentration**	**Positive valid replicates / valid replicates**	**Sensitivity %**	**Mean log_{10} titer difference EBV(+) – PSC (SD pooled)**	**Negative valid replicates / valid replicates**	**Specificity %**
No interferent (PSC)	NA	60/60	100	NA	20/20	100
NaOH	NA	60/60	100	0.02 (0.11)	20/20	100
Albumin	60 g/L	60/60	100	0.05 (0.11)	20/20	100
Bilirubin (conjugated)	342 µmol/L (0.2 g/L)	60/60	100	0.01 (0.12)	20/20	100
Bilirubin (unconjugated)	342 µmol/L (0.2 g/L)	60/60	100	0.01 (0.12)	20/20	100

Human DNA	2 mg/L	60/60	100	0.00 (0.12)	20/20	100
Hemoglobin	2 g/L	60/60	100	0.01 (0.13)	20/20	100
Triglyceride	37 mmol/L (33 ± 10* g/L)	60/60	100	0.01 (0.12)	20/20	100

*The target concentration of triglyceride is recommended by CLSI to be 33 g/L (no range stated). A concentration as low as 23 g/L is still at least five fold above the upper range reported in patients. Therefore, the results of the triglyceride testing are still relevant even at lower concentrations.

CLSI, Clinical and Laboratory Standards Institute; EBV, Epstein–Barr virus; IU, international units; LLoQ, lower limit of quantitation; PSC, positive spike control; SD, standard deviation.

Supplemental Table 8: Accuracy of the cobas EBV test in the presence and absence of microorganisms

Specimen	Interferent / pool Nr.	Valid / tested replicates	Specificity Negativity	Sensitivity	Accuracy
			Result	Result	Δ mean log10 titer
EBV (+)	1	6/6	N/A	100%	0.00
	2	6/6		100%	-0.03
	3	6/6		100%	0.04
	4	6/6		100%	-0.02
	5	6/6		100%	0.03
	6	6/6		100%	-0.03
	7	6/6		100%	-0.05
	Hepatitis C virus	6/6		100%	-0.04
	Control (no spike)	24/24		N/A	N/A
EBV (-)	1	6/6	100%	N/A	N/A
	2	6/6	100%		
	3	6/6	100%		
	4	6/6	100%		
	5	6/6	100%		
	6	6/6	100%		
	7	6/6	100%		
	Hepatitis C virus	6/6	100%		
	Control (no spike)	24/24	100%		

EBV, Epstein–Barr virus; IU, international units; LLoQ, lower limit of quantitation; PSC, positive spike control; SD, standard deviation; Δ, difference.

Supplemental Table 9: Stability of EBV-positive EDTA-plasma samples in different collection tubes, storage times, and temperature conditions

Time point	Storage temperature	Matrix and collection tube type		Mean EBV log_{10} titer	SD	Δ mean log_{10} titer (T_x – T0)
0 hours (T0 = reference)	N/A	Whole blood	Lavender top tube	2.38	0.17	N/A
	N/A	Whole blood	Plasma prep tube	2.37	0.14	N/A
24 hours (T1)	5°C ± 3°C	Whole blood	Lavender top tube	2.28	0.26	-0.10
		Whole blood	Plasma prep tube	2.30	0.18	-0.07
	25°C ± 2°C	Whole blood	Lavender top tube	2.27	0.18	-0.11
		Whole blood	Plasma prep tube	2.23	0.12	-0.15
24 hours (T2) (from whole	30°C ± 2°C	Plasma	In primary lavender top tube	2.16	0.25	-0.22
		Plasma	In primary PPT	2.24	0.15	-0.13

Time point	Storage temperature	Matrix and collection tube type		Mean EBV log_{10} titer	SD	Δ mean log_{10} titer (T_x – T0)
blood stored at 25°C)		Plasma	In secondary tube from lavender top tube collection	2.27	0.14	-0.11
		Plasma	In secondary tube from PPT collection	2.35	0.15	-0.02
6 days (T3) (from whole blood stored at 5°C)	5°C ± 3°C	Plasma	In primary lavender top tube	2.12	0.16	-0.26
		Plasma	In primary PPT	2.21	0.24	-0.16
		Plasma	In secondary tube from lavender top tube collection	2.22	0.18	-0.16
		Plasma	In secondary tube from PPT collection	2.21	0.23	-0.16
6 days (T4) (from whole	5°C ± 3°C	Plasma	In primary lavender top tube	2.15	0.28	-0.23
		Plasma	In primary PPT	2.32	0.18	-0.06

Time point	Storage temperature	Matrix and collection tube type		Mean EBV log_{10} titer	SD	Δ mean log_{10} titer (T_x – T0)
blood stored at 25°C followed by plasma stored at 30°C)		Plasma	In secondary tube from lavender top tube collection	2.27	0.21	-0.12
		Plasma	In secondary tube from PPT collection	2.26	0.16	-0.12
6 months (T7)	-20°C ± 5°C	Plasma	In secondary tube from lavender top	2.09	0.20	-0.29
		Plasma	In secondary tube from PPT collection	2.25	0.12	-0.12
	-75°C ± 15°C	Plasma	In secondary tube from lavender top	2.09	0.15	-0.29
		Plasma	In secondary tube from PPT collection	2.23	0.18	-0.14
**Freeze/thaw**						

Time point	Storage temperature	Matrix and collection tube type		Mean EBV $\log_{10}$ titer	SD	Δ mean $\log_{10}$ titer (T_x – T0)
4 freeze/thaw cycles	-20°C ± 5°C	Plasma	In secondary tube from lavender top tube collection	2.36	0.13	-0.03
		Plasma	In secondary tube from PPT collection	2.45	0.11	0.07
	-75°C ± 15°C	Plasma	In secondary tube from lavender top tube collection	2.42	0.11	0.04
		Plasma	In secondary tube from PPT collection	2.42	0.12	0.05

EBV, Epstein–Barr virus, PPT; plasma preparation tube; SD, standard deviation; T, timepoint.

References

1. Clinical and Laboratory Standards Institute. 2005. EP07-A2. Interference testing in clinical chemistry; approved guideline - second edition.
